# Clinical outcomes and anti-inflammatory mechanisms predict maximum heart rate improvement after physical activity training in individuals with psychiatric disorders and comorbid obesity

**DOI:** 10.1371/journal.pone.0313759

**Published:** 2025-01-03

**Authors:** Pau Soldevila-Matías, Joan Vicent Sánchez-Ortí, Patricia Correa-Ghisays, Vicent Balanzá-Martínez, Gabriel Selva-Vera, Roberto Sanchis-Sanchis, Néstor Iglesias-García, Manuel Monfort-Pañego, Pilar Tomás-Martínez, Víctor M. Victor, Benedicto Crespo-Facorro, Constanza San Martin Valenzuela, José Antonio Climent-Sánchez, Rosana Corral-Márquez, Inmaculada Fuentes-Durá, Rafael Tabarés-Seisdedos

**Affiliations:** 1 Faculty of Psychology, University of Valencia, Valencia, Spain; 2 INCLIVA—Health Research Institute, Valencia, Spain; 3 Department of Psychology, Faculty of Health Sciences, European University of Valencia, Valencia, Spain; 4 TMAP—Evaluation Unit in Personal Autonomy, Dependency and Serious Mental Disorders, University of Valencia, Valencia, Spain; 5 Center for Biomedical Research in Mental Health Network (CIBERSAM), Health Institute Carlos III, Madrid, Spain; 6 Teaching Unit of Psychiatry and Psychological Medicine, Department of Medicine, University of Valencia, Valencia, Spain; 7 VALSME (VALencia Salut Mental i Estigma) Research Group, University of Valencia, Valencia, Spain; 8 Department of Physical Education and Sports, University of Valencia, Valencia, Spain; 9 Department of Didactics of Physical, Artistic and Music Education, University of Valencia, Valencia, Spain; 10 Mental Health Unit of Xàtiva, Lluís Alcanys Hospital, Valencia, Spain; 11 Service of Endocrinology and Nutrition, University Hospital Dr. Peset, Valencia, Spain; 12 Foundation for the Promotion of Health and Biomedical Research in the Valencian Region (FISABIO), Valencia, Spain; 13 Department of Physiology, University of Valencia, Valencia, Spain; 14 Department of Psychiatry, University Hospital Virgen Del Rocio, IBIS-CSIC, University of Sevilla, Seville, Spain; 15 Department of Physiotherapy, University of Valencia, Valencia, Spain; 16 Mental Health Unit of Sagunto, Valencia, Spain; University Hospital of Padova, ITALY

## Abstract

**Introduction:**

This study aimed to evaluate the predictive validity and discriminatory ability of clinical outcomes, inflammatory activity, oxidative and vascular damage, and metabolic mechanisms for detecting significant improve maximum heart rate after physical activity training in individuals with psychiatric disorders and obesity comorbid using a longitudinal design and transdiagnostic perspective.

**Methods:**

Patients with major depressive disorder, bipolar disorder and, schizophrenia and with comorbid obesity (n = 29) were assigned to a 12-week structured physical exercise program. Peripheral blood biomarkers of inflammation, oxidative stress, vascular mechanisms, and metabolic activity, as well as neurocognitive and functional performance were assessed twice, before and after intervention. Maximum heart rate was considered a marker of effectiveness of physical activity. Mixed one-way analysis of variance and linear regression analyses were performed.

**Results:**

Individuals with psychiatric disorders and comorbid obesity exhibited an improvement in cognition, mood symptoms and body mass index, increase anti-inflammatory activity together with enhancement of the oxidative and cardiovascular mechanisms after physical activity training (p<0.05 to 0.0001; *d* = 0.47 to 1.63). A better clinical outcomes along with regulation of inflammatory, oxidative, and cardiovascular mechanisms were critical for predicting significant maximum heart rate variation over time (χ^2^ = 32.2 to 39.0, p < 0.0001).

**Conclusions:**

The regulation of the anti-inflammatory mechanisms may be essential for maintained of healthy physical activity across psychiatric disorders and obesity. Likewise, inflammatory activity, oxidative stress, vascular and cardio-metabolic mechanisms may be a useful to identify individuals at greater risk of multi-comorbidity.

## Introduction

Physical inactivity and sedentary behavior are significant risk factors for the development and maintained cardiovascular problems in individuals with obesity (OB) and psychiatric disorders [1, 2]. In individuals with major depressive disorder (MDD), aerobic exercise training is significantly associated with a reduction in depressive symptoms and has a positive impact on neurocognitive performance and cardiovascular health [3, 4]. Moreover, physical activity sustained over time can be an effective adjunctive treatment for the development of metabolic complications [5]. Similarly, healthier lifestyles and increased physical activity have been associated with cardiovascular gain in individuals with schizophrenia spectrum disorders and mood disorders [6, 7]. Furthermore, a prospective study also showed that physical activity is a key prognostic factor for positive trajectory bipolar disorder (BD), which reinforces its potential translation into clinical practice [8]. In individuals with schizophrenia (SZ), systematic evidence also supports this relationship [9].

A prospective study highlights that weight loss combined with high-frequency aerobic exercise has a positive impact on cardio-metabolic parameters [10]. Inflammation, oxidative damage, vascular and endothelial dysfunction are recognized as contributing factors in the common pathophysiology of OB and psychiatric disorders [11]. Likewise, chronic low-grade inflammation contributes to insulin resistance, cardiovascular risk, and joint pain, affecting healthy lifestyles and quality of life [12]. Neurocognitive and functional performance has been also linked to poorer cardio-metabolic condition. In individuals with schizophrenia and mood disorders, cognitive deficits and physical limitations may be exacerbated by vascular issues [13, 14]. Inflammatory and vascular markers contribute to decreased social and occupational functioning and diminished psychological well-being in individuals with psychiatric disorders [11]. In obesity, endothelial dysfunction contributes to reduced exercise capacity and increased fatigue, impacting functional performance [15]. Obesity, with its associated inflammation and vascular impairments, adds to physical discomfort, emotional distress, and reduced participation in social and recreational activities [12].

These molecular mechanisms play a crucial role in understanding the relationship between physical activity and cardiovascular health. Recent studies showed that blood biomarkers have been used to predict health status in individuals with psychiatric disorders and OB, including the interleukins (IL-6, IL-10), tumor necrosis factor alpha (TNF-α), and C-reactive protein (CRP) [16, 17]. Inflammation was correlated with poorer functional performance and unhealthy lifestyles [18]. Moreover, it seems that there is a close relationship between the oxidative stress and physical activity [19]. The changes in the levels of glutathione (GSH), reactive oxygen species (ROS), mitochondrial reactive oxygen species (mROS), superoxide dismutase (SOD), and mitochondrial membrane potential (ΔΨm) were associated with an unhealthy phenotype [20]. Likewise, emerging evidence suggests that endothelial dysfunction and vascular damage are associated with an increased risk of cardiovascular events in individuals with psychiatric disorder [21] and OB [22]. Fluctuations in the levels of the following cellular adhesion molecules (CAM); inter (ICAM) and vascular (VCAM) and polymorphonuclear cells (PMN); leukocyte-endothelium adhesion (LEPMN), rolling (RPMN), rolling velocity (RVPMN), pselectin (PSEL), and myeloperoxidase (MPO) were correlated with the presence of poorer physical activity [23]. Moreover, waist circumference (WC), triglycerides (TG), high- and low-density lipoprotein (HDL and LDL) blood pressure (BP), and fasting plasma glucose (FPG) could play an important role in the induction of changes in the cardiovascular health [24]. It has been suggested that a deregulations of these molecular mechanisms may be related to the cardiovascular impairment and poorer healthy status in individuals with psychiatric and OB.

In recent years, the relationship between biomarkers, clinical outcomes, inflammatory activity, oxidative and vascular damage, metabolic mechanisms, and their predictive validity for maximum heart rate has become a subject of growing interest, particularly in the context of OB and psychiatric disorders. These conditions present unique challenges, each affecting individuals’ mental health, physical well-being, and overall quality of life. Understanding how these biomarkers impact maximum heart rate can provide valuable insights for better patient care and tailored interventions. This study aims to evaluate the predictive validity of clinical outcomes, inflammatory activity, oxidative and vascular damage, and metabolic mechanisms for maximum heart rate in individuals with schizophrenia, major depression, bipolar disorder, and obesity.

## Materials and methods

The original description of the methods was elaborated by our research group [GIUV2016-312, CB/07/09/0021].

### Study design and ethical considerations

This article is part of a project aimed at finding and validating peripheral biomarkers for neurocognitive deficits in MDD, BD, SZ and T2DM carried out by Group 24 CIBERSAM (Centro de Investigación Biomédica en Red de Salud Mental) / TMAP-UV (Unidad Autonomía Personal, Dependencia y Trastorno Mental Grave—Universitat de València). This prospective and comparative cohort project was conducted between April 2015 and January 2018 to investigate the association and evolution of certain peripheral blood biomarkers and neurocognitive impairments in a unique longitudinal cohort of individuals with somatic and psychiatric disorders. Demographic and clinical data, neurocognitive and functional data, and biomarkers of peripheral blood were collected at baseline (T1) and after one year (T2). Individuals with psychiatric disorders were recruited from mental health units (MHUs) in several towns in the province of Valencia, Spain (Gandía, Foios, Catarroja, Paterna, and Sagunto); the psychiatry outpatient clinic and endocrinology department of the University Hospital Dr. Peset; and the Miguel Servet MHU in Valencia City. HCs were residents of the same areas as the individuals with psychiatric disorders. Participants were demographically matched. All participants provided informed consent after the study procedures were fully explained. The ethics committees or institutional review boards at each participating center approved the study protocol, and the study was conducted in accordance with the ethical principles of the Declaration of Helsinki. For this article, only those variables related to the present study aims were included in the analyses. This study is part of the randomized controlled trial (RCT) registered by ClinicalTrials.gov (number: NCT06069739).

### Participants

MDD, BD and SZ were diagnosed according to the criteria of the Diagnostic and Statistical Manual of Mental Disorders—DSM-5 [25]. Participants with MDD and BD should meet the remission criteria [26] of an acute affective episode, defined as Young Mania Rating Scale (YMRS) score ≤ 6 and Hamilton Rating Scale for Depression (HRSD) score ≤ 8, and individuals with SZ had to be clinically stable, defined as Positive and Negative Syndrome Scale (PANSS) score ≤ 36 [27]. The comorbid OB diagnosis was based on World Health Organization (WHO) criteria [28, 29]. For recruitment as HC, the absence of physical illness, pharmacological treatments, and family history of psychiatric disorders in first-degree relatives were required. Ability to understand study procedures and willingness to give written consent was required for participation. General exclusion criteria for all groups included: current hospitalization, documented cognitive impairment not secondary to psychiatric disorder (intellectual disability or major neurocognitive disorder, i.e. dementia), disability or inability that prevented understanding of the protocol, current substance use disorders (except for nicotine), pregnancy, intake of steroids, corticosteroids, antioxidants, antibiotics, and immunologic therapies, fever over 38°C, and history of vaccination within 4 weeks of the evaluation, medical contraindications for exercise, body mass index ≥ 40, diastolic/systolic blood pressure ≥ 140/90, resting heart rate ≥ 100. The same inclusion and exclusion criteria were used at T1 and T2.

### Intervention procedure in physical exercise

Participants were assigned to a 12-week structured physical exercise program. All treatment sessions were conducted from March to June 2016. To maintain the rigor of the research, possible risks that could question the internal and external validity of the study results were identified and strategies were proposed to eliminate them, following the indications proposed by Creswell [30].

Participants were required to attend three on-site sessions by week over 12 weeks and autonomous exercise was monitored offside the sessions through a series of weekly checkpoints. It was provided with brief healthy lifestyle counseling at baseline. To achieve the European guidelines of 150 minutes by week of moderate intensity physical activity [31], participants were prescribed three 60 minutes exercise sessions (five minutes of warm-up and five minutes of cool-down for a total of sixty minutes per session). Exercise consisted of brisk walking in urban and rural open spaces during which heart rate monitors were worn and participants were instructed to stay within their moderate intensity range (64–76% maximum heart rate (MHR) [32]. Participants gradually progressed to the target intensity and duration over the course of the first few sessions according to a gradual progression adapted to their development and personal characteristics. The standard progression was to work the first few weeks at 40–50% of MHR, increasing the intensity of the work from 50–60% MHR to 60–70% MHR in the next six weeks according to their personal development and trying to reach 70% MHR in the last weeks attending their personal level. The professional researchers followed up the sessions by conducting the initial and final phase of each session and accompanying the patients during the rest of the session in order to attend to any needs or doubts and to follow up on the fulfillment of the planned work.

### Clinical and neuropsychological assessments

The assessments were conducted by the same experienced psychologists and psychiatrists of the research group (G24 CIBERSAM/TMAP-UV). Sociodemographic data, including sex, age, years of education, premorbid Intelligence Quotient (IQ), which was calculated using the WAIS-III Vocabulary subtest, considered a classical measure of the level of intelligence before the onset of a mental disorder [33], dependent and occupational status, motor laterality (defined as manual, ocular and crural dominance), tobacco consumption, and Godin-Shephard leisure-time physical activity questionnaire (GSLTPAQ) [34], were collected.

Clinical evaluations were conducted using the following instruments: (i) the Clinical Global Impression (CGI) scale [35], (ii) 17-item Hamilton Rating Scale for Depression [36], (iii) Young Mania Rating Scale [37], (iv) Positive and Negative Syndrome Scale [38], (v) Kaplan-Feinstein Scale (KFS) [39], (vi) Charlson Comorbidity Index (CCI) [40]. The age of onset, illness duration, body mass index (BMI), total number of prescribed psychopharmacological medications and other medications were also registered.

Cognitive performance was evaluated using a comprehensive battery of neuropsychological tests and subtests previously used by our group [41–51]. Six cognitive domains were assessed: (i) *verbal learning and memory*: Complutense Verbal Learning Test (TAVEC) total immediate recall, short-term free recall and long-term free recall variables [52]; (ii) *cognitive flexibility*: Stroop Color and Word test (SCWT) color/word subtest [53] and Wisconsin Card Sorting Test (WCST) categories completed and perseverative errors [54]; (iii) *verbal fluency*: FAS and animal naming test for phonemic and semantic fluency, respectively [55]; (iv) *working memory*: Trail Making Test (TMT) Part B [55] and Wechsler Adult Intelligence Scale III edition (WAIS-III) digit span backwards [56]; (v) *short-term memory*: TAVEC immediate recall of the first learning trial and immediate recall of the interference list [52] and WAIS-III digit span forward [56]; (vi) *visual memory*: Rey-Osterrieth Complex Figure Test (ROCFT) figure two minutes after the copy (fRey2) and 20 minutes after the copy (fRey20) [57]; and (vii) *processing speed*: finger tapping test (FTT) left unimanual, right unimanual, left bimanual, right bimanual and average four scores [55, 58], WAIS-III digit symbol coding subtest [56], SCWT color and word subtests [53] and TMT Part A [55]. A global cognitive score (GCS) was calculated by averaging the seven cognitive domain scores.

Functional performance was evaluated using: (i) the Functional Assessment Short Test (FAST) [59], (ii) the Short Form-36 Health Survey questionnaire (SF-36) [60], and (iii) the World Health Organization Quality of Life brief scale (WHO-QoL-Bref) [61]. A global functional score (GFS) was calculated by averaging the total scores on the three scales.

### Determination of biomarkers in peripheral blood

Venesection was performed, and serum and plasma samples were stored in a freezer at –80°C.

### (i) Inflammatory markers

Serum cytokine concentrations were determined using Luminex® X-MAP technology (Luminex Corp., Austin, TX, USA) based on flow cytometry. The following cytokines were analyzed: interleukins (IL-6 and IL-10), and tumor necrosis factor alpha (TNF-α). Sample processing and data analysis were performed according to the manufacturer’s instructions. C-reactive protein (CRP) levels were determined using an immunonephelometric assay (Behring Nephelometer II, Dade Behring, Inc., Newark, DE, USA).

### (ii) Oxidative stress markers

Oxidative stress in leukocytes was evaluated using fluorimetry techniques with a fluoroscan (Synergy MX). In total, 100 000 cells were plated in each well of 96-well plates and incubated for 30 min at 37°C with the corresponding fluorochromes, as follows: dichlorofluorescein diacetate to measure reactive oxygen species (ROS) production (485 nm excitation, 535 nm emission), MitoSOX to measure mitochondrial ROS (mROS) (510 nm excitation, 580 nm emission), tetramethylrodamin methyl ester to assess mitochondrial membrane potential (ΔΨm) (552 nm excitation, 574 nm emission), superoxide dismutase (SOD) (total SOD/T-SOD Activity Assay Kit–Colorimetric—from NOVUS), and 5-chloromethylfluorescein diacetate to measure intracellular glutathione (GSH) (492 nm excitation, 517 nm emission). The monocyte cell line U-937 was used as an internal control to avoid potential fluctuations in fluorescence over time.

### (iii) Adhesion molecules

Serum lipid peroxidation levels were measured using a commercial thiobarbituric acid reactive substances (TBARS) kit according to the manufacturer’s instructions (Olympus, Hamburg, Germany). A Luminex 200 flow analyzer system (Austin, TX, USA) was employed to analyze cellular adhesion molecules (CAM) in serum. To measure immunological markers, citrated blood samples were incubated with dextran (3%) for 45 min to isolate human polymorphonuclear leukocytes (PMNs). The supernatant was layered over Ficoll-Hypaque (GE Healthcare, Barcelona, Spain) and centrifuged for 25 min at room temperature at 650g. Lysis buffer was added to the remaining erythrocytes in the pellet, which were incubated at room temperature for 5 min and then spun at 240g for 5 min. PMNs were rinsed twice and resuspended at 37° in Hanks’ balanced salt solution (Sigma Aldrich, MO). Scepter 2.0 cell counters (Millipore, MA, USA) was employed to count cells.

PMNs were isolated as previously described. 56 A 1.2-mL aliquot of PMNs was obtained from the peripheral blood of HCs and patients at a density of 106 cells/mL in complete RPMI (RPMI 1640 medium supplemented with 10% fetal bovine serum, 1% penicillin/streptomycin, 1% glutamine, and 1% sodium pyruvate). Prior to this, primary cultures of human umbilical cord endothelial cells (HUVECs) were established. HUVECs were isolated as previously reported. On the day of experimentation, PMNs were monitored through the endothelial monolayer at a speed of 0.3 mL/min over a 5-min period. Activity was recorded, and the number, velocity, and adhesion to the endothelial monolayer of rolling PMNs were determined. The number of rolling PMNs was measured as those rolling for 1 minute. Velocity was assessed by determining the time in which 15 rolling PMNs covered 100 μm. Adhesion was analyzed by counting the number of PMNs adhering to the endothelium for at least 30 s in five fields.

### (iv) Cardio-metabolic markers

The following cardio-metabolic markers were collected as follows: waist circumference (WC) (cm), triglycerides (TG), high-density lipoprotein (HDL), low-density lipoprotein (LDL), systolic blood pressure (SBP) / diastolic blood pressure (DBP) (mmHg), fasting plasma glucose (FPG) and maximum heart rate (MHR). The MHR was measured with a watch-shaped device that was worn on the wrist and captured the beats per minute. Body weight, height, and WC were measured by calibrated scales. WC was measured in the standing position at the end of normal expiration and at the midway between the inferior costal margin and the superior border of the iliac crest. BP was measured on the right arm using an automatic sphygmomanometer with participants in the sitting position after resting for 5 minutes. Average SBP and DBP values of at least two repeated measurements were calculated. Under aseptic conditions, fasting venous blood samples were collected between 8 and 9 am to measure TG, LDL, HDL and FPG levels. Individuals with diseases followed the prescribed pharmacological treatment throughout the study.

### Statistical analyses

Data were analyzed using Statistical Package for Social Sciences (SPSS) version 26.0 for Windows [62]. The sample size was calculated using Ene 2.0 software, which estimated that twenty-nine individuals were sufficient to ensure the representativeness [63]. Descriptive analyses were expressed as mean (standard deviation) for continuous variables and total number (percentage) for categorical variables. Normality was assumed for all continuous variables because the sample was statistically verified using Shapiro-Wilk test. This fact guarantees that the variables were distributed in a normalized way. The differences between times for clinical characteristics and biomarkers were assessed using a t-test for dependent samples. To test predictive capacity the clinical outcomes and biomarkers at T1 to explain the variance of change maximum heart rate before and after of the physical activity training, a linear regression analysis was performed using a predictive model that included all variables that were significant. To test the ability of clinical outcomes and biomarkers at T1 to discriminate between individuals with improve maximum heart rate, a discriminant analysis was performed used all baseline variables. Subsequently, a single model using only significant clinical characteristic and biomarkers was tested. For all analyses, p < 0.05 was considered statistically significant. The effect size was calculated with Cohen’s d (d) and the following values were taken as reference: small ≈ 0.20; moderate ≈ 0.50; large ≈ 0.80.

## Results

### Sample description

At T1, the sample consisted of 29 persons with psychiatric disorders, including 17 with SZ, 6 with BD, and 6 with MDD, and comorbid diagnosis of obesity (OB). None participants were lost to follow-up at T2 (retention rate: 100%).

Females represented 31% of the total sample. The mean age was 47.3 (SD: 10.2) years and 10.0 (SD: 3.7) years of education of the whole sample. It was characterized by IQ standard values 96.2 (SD: 15.5) and moderately active physical condition 41.6 (SD: 39.4). The entire sample was unemployed (100%) and less than half were dependent (38%). Moreover, the great majority of the sample was right-handed (90%) and approximately half of the individuals used tobacco (52%).

### Between-group comparison of clinical outcomes and biomarkers

Clinical characteristics at both times are shown in **Table 1**. The individuals had an age of illness onset close to 25 years and mean illness duration of 22 years. Concerning to mental health outcomes, individuals showed significant improvement in cognition (p < 0.0001; *d* = 0.87) and mood symptoms (p < 0.01; *d* = 0.53) after the physical activity training was performed. Likewise, with regard to physical health outcomes, a decrease in BMI was observed over time (p < 0.01; *d* = 0.64). In all cases, the effect size was from moderate to large.

**Table 1:** Clinical characteristics.

Variables^a^	T1	T2	*Statistical analyses*	
	T1 (*n* = 29)	T2 (*n* = 29)	t(*p*)^f^	*d* ^g^
***Mental health outcomes***				
**Age of onset** ^**b**^	25.6(8.6)	-		
**Illness duration** ^**b**^	22.2(10.9)	-		
**CGI** ^**c**^	4.5(1.1)	4.3(1.3)	.24	
**HRSD** ^**c**^	9.7(5.4)	9.8(6.3)	.93	
**YMRS** ^**c**^	6.1(5.3)	3.5(4.4)	.01	.53
**PANSS-P** ^**c**^	12.1(6.3)	11.1(6.6)	.11	
**PANSS-N** ^**c**^	16.5(10.5)	17.2(10.2)	.52	
**PANSS-G** ^**c**^	33.6(14.7)	33.0(15.7)	.68	
**Psychiatric medicines** ^**d**^	4.7(2.3)	4.8(2.2)	.76	
**GCS**	238.3(166.8)	350.2(165.6)	< .0001	.87
***Physical health outcomes***				
**BMI**	32.0(4.0)	31.6(4.1)	.01	.64
**KFS**	0.6(1.0)	0.5(0.8)	.16	
**CCI**	0.5(0.9)	0.4(0.6)	.29	
**Tobacco** ^**e**^	10(34.5)	10(34.5)	.32	
**General medicines** ^**d**^	5.0(2.3)	5.1(2.2)	.65	
**GFS**	2247.5(533.4)	2105.7(664.3)	.12	

^a^ Expressed as mean (standard deviation) except when indicated

^b^ years

^c^ lower scores represent a better outcome

^d^ number

^e^ yes n (%)

^f^ paired t-test for dependent samples

^g^ Cohen’s d. Abbreviations: T1 = time 1, T2 = time 2, CGI = clinical global impression, HRSD = Hamilton rating scale for depression, YMRS = Young mania rating scale, PANSS = positive and negative syndrome scale, P = positive, N = negative, G = general, GCS = global cognitive score, BMI = body mass index, KFS = Kaplan-Feinstein scale, CCI = Charlson comorbidity index, GFS = global functional score. Effect size (*d*: small ≈ 0.20; moderate ≈ 0.50; large ≈ 0.80).

Peripheral serum markers at both times are shown in **Table 2**. Regarding the inflammatory state, anti-inflammatory biomarker IL-10 had significantly increased after the physical activity training was carried out (p < 0.05; *d* = 0.47). Similarly, for oxidative stress markers, ROS had significantly higher concentrations after of the physical activity training was observed (p < 0.01; *d* = 1.63) and, by contrast, mROS had significantly lower concentrations after of the physical activity training (p < 0.01; *d* = 0.56). Moreover, LEPMN marker significantly decreased (p < 0.01; *d* = 0.46) and insulin sensitivity had significantly higher levels after the physical activity training (p < 0.01; *d* = 0.56). It should be noted that MHR significantly decreased after the physical activity training (p < 0.01; *d* = 0.49). In all cases, the effect size was from moderate to large.

**Table 2:** Biomarkers.

Variables^a^	T1	T2	*Statistical analyses*	
	(*n* = 29)	*(n* = 29)	t(*p*)^b^	*d* ^c^
***Inflammatory markers***				
**IL-6**	2.8(2.0)	2.6(2.6)	.52	
**IL-10**	45.6(42.3)	60.1(49.2)	.02	.47
**TNF-α**	9.0(2.6)	9.6(4.7)	.31	
**CRP**	4.7(4.8)	7.3(9.3)	.16	
***Oxidative stress markers***				
**GSH**	166.3(106.5)	173.4(74.7)	.76	
**ROS**	106.2(26.0)	161.8(88.8)	.002	1.63
**mROS**	187.6(81.7)	131.0(26.0)	.003	.56
**SOD**	102.8(17.3)	96.7(14.9)	.11	
**ΔΨm**	40.3(22.1)	44.1(17.3)	.48	
***Adhesion molecules***				
**ICAM**	118.0(45.7)	123.4(58.6)	.59	
**VCAM**	591.9(126.3)	603.5(142.0)	.56	
**LEPMN**	11.1(8.0)	6.0(2.8)	.003	.46
**RPMN**	231.3(105.4)	208.1(65.9)	.24	
**RVPMN**	411.1(134.9)	431.2(98.3)	.46	
**PSEL**	132.3(28.8)	135.7(38.9)	.64	
**MPO**	725.6(427.8)	755.4(418.2)	.62	
***Cardio-metabolic markers***				
**WC**	109.0(12.1)	108.3(10.4)	.44	
**TG**	178.2(62.2)	179.4(80.6)	.91	
**HDL**	43.6(11.7)	43.9(12.4)	.79	
**LDL**	116.6(43.5)	114.9(30.0)	.72	
**SBP**	121.9(12.8)	123.0(11.5)	.54	
**DBP**	78.3(10.1)	79.3(9.4)	.23	
**FPG**	97.8(18.3)	97.3(18.7)	.76	
**Insulin**	16.4(10.4)	22.2(13.5)	.01	.56
***Physical exercise effectiveness marker***				
**MHR**	184.9(23.7)	172.5(10.2)	.004	.49

^a^ Expressed as mean (standard deviation)

^b^ paired t-test for dependent sample

^c^ Cohen’s d. Abbreviations: T1 = time 1, T2 = time 2, IL-6 = interleukin-6, IL-10 = interleukin-10, TNF-α = tumor necrosis factor alpha, CRP = c-reactive protein, GSH = glutathione, ROS = reactive oxygen species, mROS = mitochondrial reactive oxygen species, SOD = superoxide dismutase, ΔΨm = mitochondrial membrane potential, CAM = cellular adhesion molecule, PMN = polymorphonuclear cells, I = inter, V = vascular, LE = leukocyte-endothelium adhesion, R = rolling, RV = rolling velocity, PSEL = pselectin, MPO = myeloperoxidase, WC = waist circumference, TG = triglycerides, HDL = high-density lipoprotein, LDL = low-density lipoprotein, SBP = systolic blood pressure, DBP = diastolic blood pressure, FPG = fasting plasma glucose, MHR = maximum heart rate. Effect size (*d*: small ≈ 0.20; moderate ≈ 0.50; large ≈ 0.80).

### Predictive capacity of clinical outcomes and biomarkers at T1 of change maximum heart rate before and after the physical activity training

The results of the relative contributions of clinical outcomes and biomarkers at T1 to explain the variation of MHR were shown in **Table 3**. The combination of clinical outcomes (age of onset and illness duration), anti-inflammatory activity (IL-10), mitochondrial membrane potential (ΔΨm), and cardio-metabolic parameter (FPG) significantly predicted MHR over time and explained 41.2% of the variance. Similarly, the combination of clinical outcomes (PANSS-N), anti-inflammatory activity (IL-10), oxidative stress (ROS), leukocyte-endothelium interactions (rolling polymorphonuclear cells [RPMN]), and pselectin [PSEL]) significantly predicted MHR over time and explained 48.0% of the variance. Clinical outcomes (HRSD), GFS, and adhesion molecules also significantly predicted MHR over time and explained 59.1% of the variance. Moreover, clinical outcomes (HRSD), oxidative stress biomarker (SOD), and adhesion molecules (vascular cellular adhesion molecule [VCAM], leukocyte-endothelium polymorphonuclear cells [LEPMN] and rolling velocity polymorphonuclear cells [RVPMN]) significantly predicted MHR over time and explained 67.7% of the variance. Lastly, the combination of clinical outcomes (PANSS-G), inflammatory activity (CRP), oxidative stress (ROS), and adhesion molecules (LEPMN and RVPMN) significantly predicted MHR over time and explained 72.1% of the variance. There was a negative relationship between clinical outcomes (p < 0.01 –p < 0.0001), oxidative stress (p < 0.05 –p < 0.0001), vascular damage (p < 0.01 –p < 0.0001), GFS (p < 0.05), and pro-inflammatory activity (p < 0.05), whereas a positive correlation with anti-inflammatory mechanisms (p < 0.05 –p < 0.01) associated with MHR variation for all predictive models.

**Table 3:** Predictive clinical outcomes and biomarkers at T1 of change maximum heart rate.

Dependent variables at T2	Predictors at T1	*β*	95% CI	*p*	Percent of variance explained (adjusted *R*^*2*^)
**MHR (T2-T1)**	**Age of onset**	-.70	-2.79 to -.67	.003	41.2
	**Illness duration**	-.60	-2.04 to -.30	.01	
	**IL-10**	.39	.04 to .35	.01	
	**ΔΨm**	-.52	-.86 to -.14	.008	
	**FPG**	.38	.02 to .87	.03	
	**PANSS-N**	-.58	-1.82 to -.53	.001	48.0
	**IL-10**	.33	.01 to .31	.03	
	**ROS**	-.27	-.46 to .02	.07	
	**RPMN**	.40	.01 to .14	.01	
	**PSEL**	.35	.04 to .48	.02	
	**HRSD**	-.61	-3.58 to -1.19	< .0001	59.1
	**GFS**	-.32	-.02 to -.001	.04	
	**ICAM**	.27	.001 to .25	.04	
	**RVPMN**	-.52	-.12 to -.03	.001	
	**HRSD**	-.49	-2.8 to -1.0	< .0001	67.7
	**SOD**	-.46	-.89 to -.25	.001	
	**VCAM**	.28	.008 to .08	.02	
	**LEPMN**	-.32	-1.48 to -.22	.01	
	**RVPMN**	-.65	-.14 to -.06	< .0001	
	**PANSS-G**	-.53	-1.09 to -.46	< .0001	72.1
	**CRP**	-.23	.10 to 1.97	.03	
	**ROS**	-.33	-.44 to -.10	.003	
	**LEPMN**	-.26	-1.26 to -.12	.01	
	**RVPMN**	-.47	-.10 to -.04	< .0001	

Abbreviations: T1 = time 1, T2 = time 2, MHR = maximum heart rate, IL-10 = interleukin-10, CRP = c-reactive protein, ROS = reactive oxygen species, SOD = superoxide dismutase, ΔΨm = mitochondrial membrane potential, CAM = cellular adhesion molecule, PMN = polymorphonuclear cells, I = inter, V = vascular, LE = leukocyte-endothelium adhesion, R = rolling, RV = rolling velocity, PSEL = pselectin, FPG = fasting plasma glucose, GFS = global functional score, HRSD = Hamilton rating scale for depression, PANSS = positive and negative syndrome scale, N = negative, G = general.

### Discriminatory ability of clinical outcomes and biomarkers on improvements in maximum heart rate

**Fig 1:**
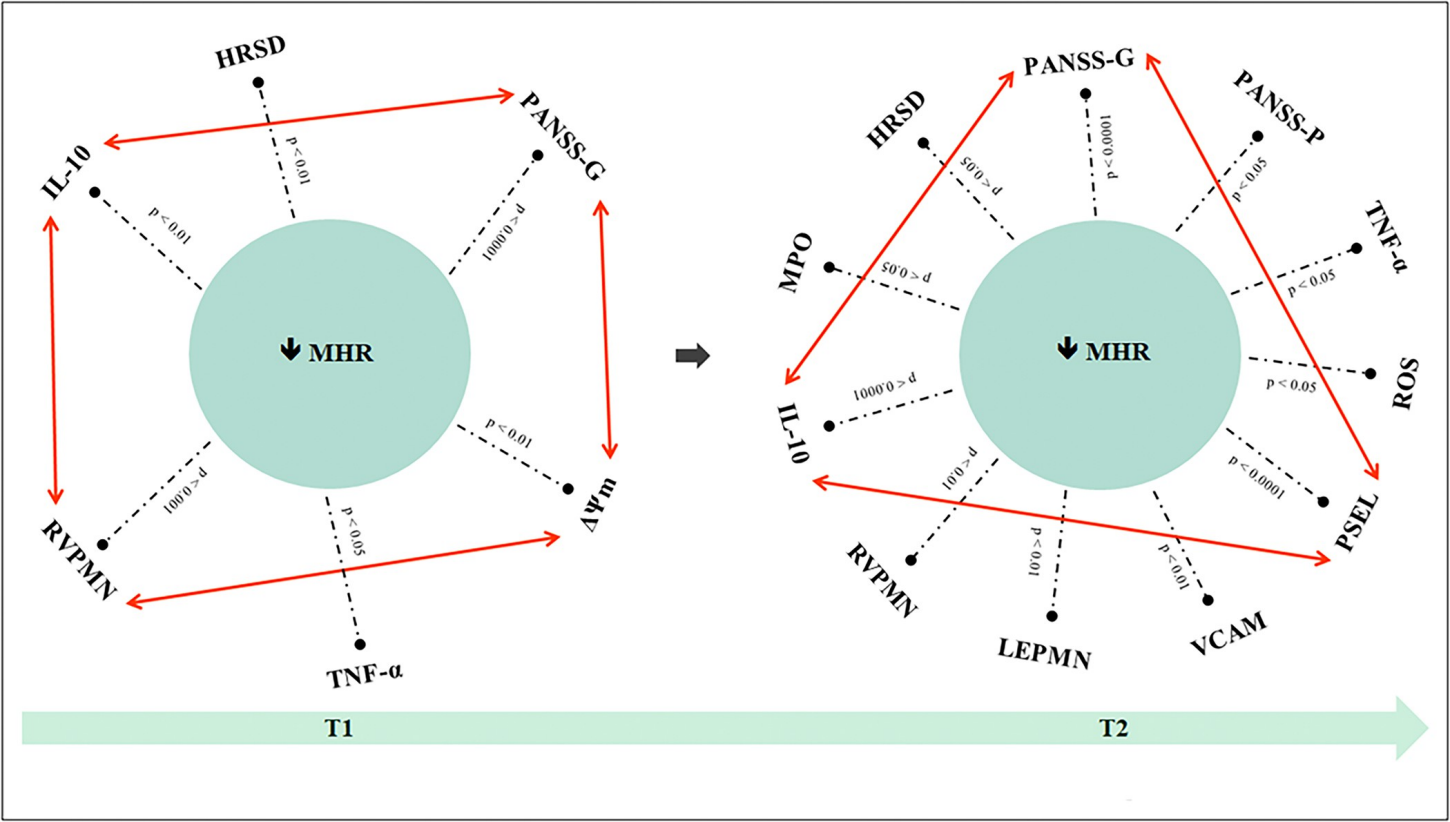
Discriminatory capacity of the clinical outcomes and biomarkers to differentiate to the individuals with improved MHR. MHR: maximum heart rate, HRSD: Hamilton rating scale for depression, PANSS-P: positive and negative syndrome scale (positive subscale), PANSS-G: positive and negative syndrome scale (general subscale), IL-10: interleukin 10, TNF-α: tumor necrosis factor alpha, ROS: reactive oxygen species; ΔΨm: mitochondrial membrane potential, VCAM: vascular cellular adhesion molecule, LEPMN: leukocyte-endothelium adhesion polymorphonuclear, RVPMN: rolling velocity polymorphonuclear cells, PSEL: P-selectin; MPO: myeloperoxidase.

The results regarding the discriminatory ability of clinical outcomes and serum peripheral markers showed that the combination of PANSS-G, IL-10, RVPMN, ΔΨm was the transdiagnostic model that best discriminated between individuals with improved maximum heart rate at T1 (χ^2^ = 32.2, p < 0.0001). Likewise, the combination of PANSS-G, IL-10, PSEL was the transdiagnostic model that best discriminated between individuals with improved MHR at T2 (χ^2^ = 39.0, p < 0.0001) (**Fig 1**). According to the models, individuals with improved MHR were characterized by more severe general psychopathology, increased rolling velocity, and larger mitochondrial membrane potential, while lower anti-inflammatory activity at T1. In contrast, these same individuals were characterized by lower general psychopathology, and better anti-inflammatory mechanisms at T2.

## Discussion

To the best of our knowledge, this study is the first to examine the predictive validity of clinical outcomes such as inflammatory activity, oxidative and vascular damage, and metabolic mechanisms concerning maximum heart rate as a marker for physical activity effectiveness. Additionally, we evaluated their ability to discriminate significant improvements in maximum heart rate following physical activity training in individuals with psychiatric disorders and comorbid obesity. Our study employs a longitudinal design and adopts a transdiagnostic perspective.

The present results show that individuals with psychiatric disorders and OB comorbid exhibited an improvement in cognition, mood symptoms, BMI, and increased anti-inflammatory activity together with enhancement of the oxidative and cardiovascular mechanisms after physical activity training. Indeed, better clinical outcomes (age of onset and illness duration, HRSD, and PANSS) along with regulation of inflammatory (IL-10 and CRP), oxidative (ΔΨm, ROS, and SOD) and cardiovascular (i.e. LEPMN, RPMN, and RVPMN) mechanisms were critical for predicting significant MHR variation over time. Moreover, the discriminant analysis showed that general psychopathology and anti-inflammatory activity were able to distinguish individuals with improved MHR.

Our findings are in accordance with previous evidence suggesting that physical activity sustained over time improves neurocognitive performance and mood symptoms across psychiatric disorders [64]. In individuals with OB, meta-analytic evidence also supports this relationship [65]. Likewise, having an established physical exercise routine has favorable effects on BMI and other weight parameters in these individuals [66, 67]. Recent findings suggest that exercise induces an anti-inflammatory effect, which positively impacts the immune system response [68]. Specifically, IL-10´s anti-inflammatory action has been postulated as potential common therapeutic avenue linked to physical exercise, as it promotes stabilization of inflammatory parameters across chronic pathologies [69]. Our results indicate that oxidative stress is involved in the beneficial effects of exercise, aligning with findings from recent studies [70]. Moderate levels of exercise-induced ROS production play an essential role in promoting physiological benefits, such as enhancing immunological function [19]. Additionally, improved mitochondrial ROS capacity provides a beneficial adaptive effect on the cellular antioxidant system, potentially preventing neurodegenerative diseases associated with impaired mitochondrial recycling [71]. Moreover, improvement of cardio-metabolic parameters is directly related to increased physical activity. Growing evidence supports that leukocyte-endothelium adhesion is significantly reduced with the continued practice of physical exercise and was strongly related to improving healthy lifestyles [72]. Exercise also improves insulin sensitivity through the reduction of cytokines, inflammatory and oxidative stress responses [73]. Likewise, exercise decreases nicotine consumption in individuals with psychiatric disorders and promotes a toxic-free environment that is conducive to a healthy lifestyle. In fact, reducing nicotine levels has a positive impact on inflammation and reduces cardiovascular risk [74].

Consider removing and promote a toxic-free environment that is conducive to a healthy lifestyle.

Exercise has been identified as a promising intervention to improve physical and mental health outcomes in individuals with comorbidities, positioning itself as a key element in reducing cardiovascular risk [75]. In accordance with our findings, existing evidence indicates a strong relationship between the severity of mood and psychotic symptoms and cardiovascular activity. This connection is thought to be mediated by underlying biological mechanisms related to the increase of inflammatory markers and autonomic nervous system dysregulation [76, 77]. Similarly, our findings align with existing evidence demonstrating a critical link between inflammatory activity and heart rate performance. We postulate that cardiovascular activity may adaptively regulate inflammatory processes [78]. Our results converge also with previous findings suggesting that ROS and mROS generation mediate the relationship between clinical severity and HR performance, and it is associated with poorer outcomes in physical activity [79]. Similarly, endothelial dysfunction is associated with worse prognosis and higher rate of cardiovascular events [80]. Thus, a better understanding of the mitochondrial and vascular endothelial adaptations to exercise can shed light on the mechanisms of exercise-induced cardiovascular protection and provide new tools to orient precise exercise therapies for mental and physical health [81, 82]. Indeed, recent studies show that HR monitoring could be used as a clinical marker to identify increased risk of developing inflammation and cardiovascular damage in people with OB [83] and that vagal stimulation and anti-inflammatory treatments can be key factors for support the pharmacological therapy in individuals with MDD [84]. In fact, it has been observed that heart rate can be a potentially powerful transdiagnostic biobehavioral change mechanism associated with several psychiatric disorders [85] and OB [86]. It should be noted that advanced understanding of this relationship may be useful to maximize the benefits of regular exercise on psychopathology and functional impairment [87] and promote and promote the importance of clinicians being aware of the benefits of exercise for these conditions [88].

Our study has several limitations and strengths. Firstly, the sample was relatively small and was composed of individuals with psychiatric disorders and OB comorbid only; however, the multicenter design of the study may increase the external validity of the results and compensate for such limitation. Second, it is a prospective study from which data was gathered over two times, allowing for more robust cause-effect relationships to be established between clinical variables, biomarkers and heart rate. It should be noted that retention rate was at a maximum. Third, while we included many molecular parameters thought to affect heart rate, there may be additional variables not included in our study that may show an association. Moreover, inflammatory biomarkers interact in a complex manner with other parameters, such as oxidative stress, vascular activity, and cardio-metabolic mechanisms to mediate inflammation which limits the interpretation of our findings. However, we believe that the variables we included are the most common serum parameters that have clinical relevance. Fourth, our analysis may come with potential biases including ascertainment, disease classification, and sample selection bias, which may affect overall internal validity. However, we did controlee the significant socio-demographic variables that could affect to internal validity, making this issue less outstanding. Fifthly, while brief counseling on healthy lifestyle was provided at baseline, we did not assess lifestyle behaviors beyond physical activity in this study [89, 90]. Factors such as hydration, nutritional intake, sleep patterns, and pre-workout preparation could also influence maximum heart rate and overall cardiovascular performance, warranting further investigation. Lastly, it is important to mention that peripheral blood biomarkers among the individuals with psychiatric disorders and comorbid OB can be fluctuate depending on the stage of the mental disorder or BMI, as well as pharmacological and lifestyle interventions [91].

## Conclusion

In conclusion, this study emphasizes the key role of molecular mechanisms in the exercise across several disorders. Our findings suggest that maintaining good mental health and properly regulating inflammatory, oxidative, and cardiovascular mechanisms are essential for sustaining healthy physical activity levels. This interplay underscores the importance of exercise in enhancing mental health and modulating biological systems. Specifically, our findings support that biomarker-based model can be considered as a potential pathway for discerning those individuals with the greatest potential to benefit from physical exercise across pathologies. If confirmed by future studies, this model could facilitate a more personalized application of exercise that will implement the benefits of physical activity from early stages and enhance healthy lifestyles in order to prevent future complications linked to the course of the disease. Therefore, our study highlights the potential of using biomarkers as predictors for personalized exercise interventions in individuals with psychiatric disorders and comorbid obesity. By leveraging these insights, clinicians can develop tailored exercise programs to maximize heart rate improvements, ultimately contributing to enhanced physical and mental health outcomes. The results support the use of specific biomarkers as valuable tools for predicting the effectiveness of exercise interventions, thereby guiding personalized treatment strategies. It will enable us to move forward in our understanding of the impact of molecular mechanisms and psychopathology on physical exercise in individuals with comorbidities from a transdiagnostic perspective.

## Supporting information

S1 Data (XLSX)
